# Possible bite-induced abscess and osteomyelitis in *Lufengosaurus* (Dinosauria: sauropodomorph) from the Lower Jurassic of the Yimen Basin, China

**DOI:** 10.1038/s41598-018-23451-x

**Published:** 2018-03-22

**Authors:** Lida Xing, Bruce M. Rothschild, Patrick S. Randolph-Quinney, Yi Wang, Alexander H. Parkinson, Hao Ran

**Affiliations:** 1 State Key Laboratory of Biogeology and Environmental Geology, China University of Geosciences, Beijing, 100083 China; 2 School of the Earth Sciences and Resources, China University of Geosciences, Beijing, 100083 China; 3 Division of Vertebrate Paleontology, Carnegie Museum of Natural History, 4400 Forbes Avenue, Pittsburgh, PA 15271 USA; 4 West Virginia University School of Medicine, Morgantown, WV 26506 USA; 5 School of Forensic and Applied Sciences, University of Central Lancashire, Preston, PR1 2HE UK; 6 Evolutionary Studies Institute, University of the Witwatersrand, Wits, 2050 Johannesburg, South Africa; 7 School of Anatomical Sciences, University of the Witwatersrand Medical School, Johannesburg, South Africa; 8 Yuxi Museum, Yunnan, 653100 China; 9 School of Geosciences, University of the Witwatersrand, Wits, 2050 Johannesburg, South Africa; 10 State Key Laboratory of Genetic Resources and Evolution, Kunming Institute of Zoology, Chinese Academy of Sciences, Kunming, 650223 China; 11 Key Laboratory of Ecology of Rare and Endangered Species and Environmental Protection, Ministry of Education, Guilin, 541004 China

## Abstract

We report an osseous abnormality on a specimen of the sauropod dinosaur *Lufengosaurus huenei* from the Fengjiahe Formation in Yuxi Basin, China. A gross pathological defect occurs on the right third rib, which was subjected to micro-computed tomographic imaging as an aid in diagnosis. The analysis of pathological characteristics and the shape of the abnormality is incompatible with impact or healed trauma, such as a common rib fracture, and instead suggests focal penetration of the rib, possibly due to a failed predator attack. The identification of characteristics based on gross morphology and internal micro-morphology presented by the specimen, suggests an abscess with osteomyelitis as the most parsimonious explanation. Osteomyelitis is a severe infection originating in the bone marrow, usually resulting from the introduction of pyogenic (pus-producing) bacteria into the bone. Micro-tomographic imaging of the lesion suggests a degree of healing and bone remodelling following post-traumatic wound infection with evidence of sclerotic bone formation at the site of pathological focus, indicating that *L. huenei* survived the initial trauma. However, as osteomyelitis can express through widespread systemic effects, including a lowering of immune response and overall condition, this disease may have been a contributing factor to the eventual death of the individual.

## Introduction

We report evidence for pathological lesions in a partial skeleton of *Lufengosaurus huenei* from the Fengjiahe Formation in Yuxi Basin, Yunnan Province, China, which may be attributed to a failed predator attack on this sauropod. The analysis of osseous abnormalities can provide critical insight into palaeo-immunology, behavioural and life history information for dinosaurs, as well as environmental insights into ancient ecosystems^1–3^. A modern understanding of forensic trauma analysis and pathology, as well as pathologies in modern birds, reptiles and mammals provides a platform for identification of similar palaeopathologies in the fossil record. The fundamental principle underlying the inference of palaeopathological conditions from modern comparative analysis is that of uniformitarianism^4^, and this approach has yielded significant data regarding the expression and response to disease and trauma in the dinosauria. In particular, the fossil record of China has yielded a range of reports of palaeo-bone pathologies including healed bite marks in *Sinraptor*^5^, bacterial infection in the fibula of the basal ceratopsian *Psittacosaurus*^6^, a healed fracture in the theropod *Yangchuanosaurus*^7^, osteoarthritis in the theropods *Caudipteryx*, *Confuciusornis* and *Microraptor*^8^, tooth loss and alveolar remodeling in *Sinosaurus triassicus*^9^, and vertebral fusions in sauropodomorph dinosaurs^1^. The specimen reported here adds evidence of both bone-pathology, as well as inferred behaviour, to this growing hypodigm of skeletal pathologies.

## Geological setting

The Fengjiahe Formation is the oldest Lower Jurassic red bed unit in the region and varies in thickness throughout^10^. The formation is stratigraphically positioned between the Upper Middle Jurassic Zhanghe Formation, and the Lower Triassic Shezi Formation. The Fengjiahe Formation is 1500 m thick in some regions, and its upper contact with the Shezi formation is conformable^10^. The formation is characterised by purple-red mudstone and argillaceous siltstone interbedded with gray-green and yellow-green quartz sandstone and feldspathic quartz sandstone. The formation is also associated with a rich track-bearing horizon.

The Fengjiahe Formation has yielded a diversity of vertebrate fossils and is renowned for the *Lufengosaurus* fauna, consisting mostly of the basal sauropodomorphs, *Lufengosaurus* and *Yunnanosaurus*^11^. The fauna recovered from this formation is comparable with fauna from the Lufeng Formation^12^. The Lufeng Formation is the most famous and prolific unit of the Mesozoic strata of Yunnan Province in China. This formation contains a rich vertebrate fauna, including dinosaurs (sauropodomorph, theropod and ornithischian), early mammals and non-mammalian eucynodonts, basal crocodylomorphs, and sphenodontians^13–17^.

## Materials and Methods

**Figure 1:**
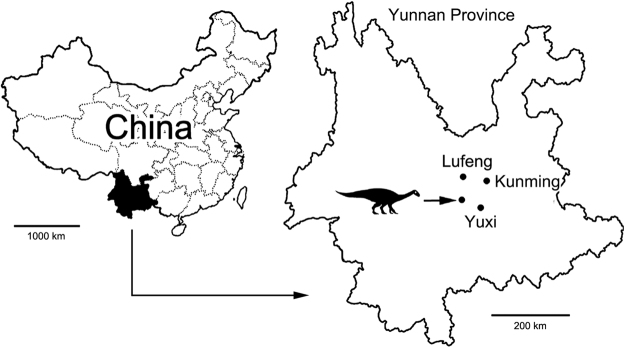
Map showing the position of the *Lufengosaurus huenei* dinosaur fossil locality in Yunnan Province, China. Line drawing modified from Xing and colleagues^1^.

The specimen analysed during the course of this study was recovered from the Yuxi Basin, Yunnan Province, China (Fig. 1) and is housed at Yuxi Museum, Yunnan, under accession number YX V0003. The specimen was referred to *Lufengosaurus huenei* based on the manus being longer than the ulna and the pubis-illium length ratio of 1.1^18–21^. YX V003 comprises a partial articulated skeleton which includes 9 cervical vertebrae, 10 dorsal vertebrae, 3 sacral vertebrae, 7 caudal vertebrae, scapulas, humeri, hip bones, and limb bones. The specimen was recovered from the No.3 bonebed, Jiaojiedian Village, Shijie Township, Yimen County, Yunnan Provence, China. The entire specimen was inspected morphoscopically for any evidence of paleopathological lesions.

**Figure 2:**
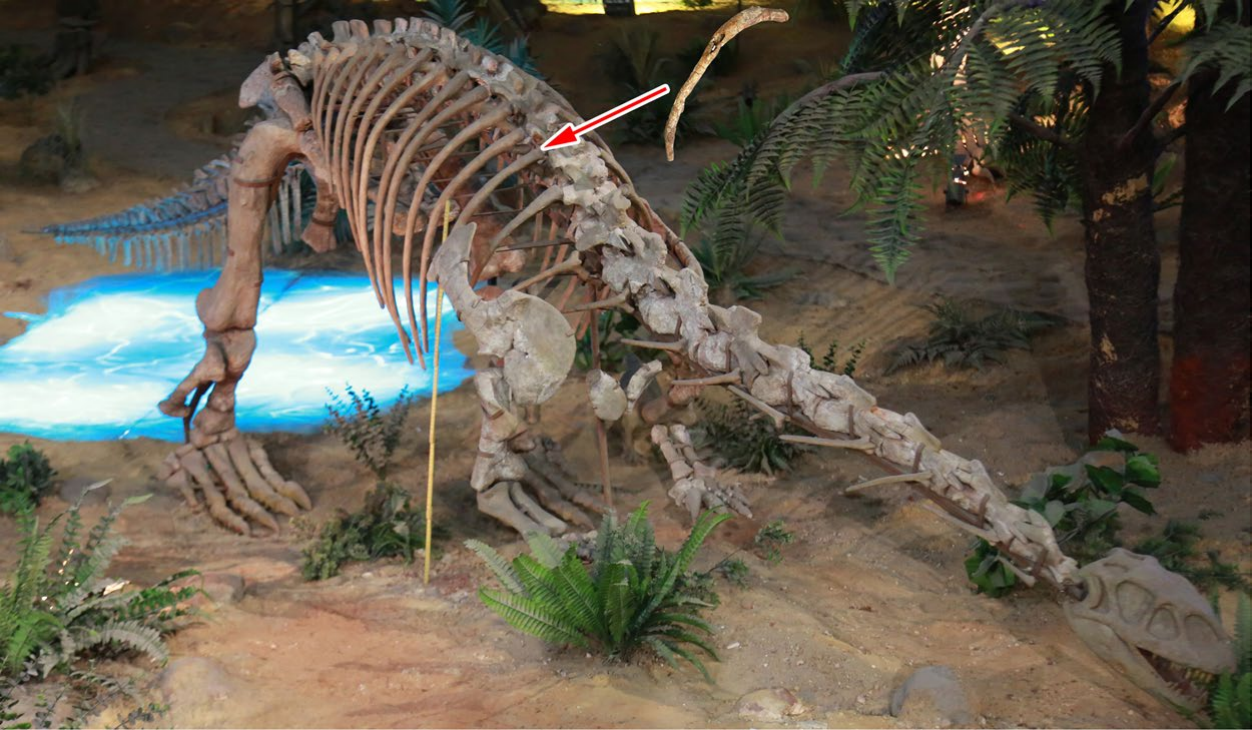
Museum reconstruction of the *Lufengosaurus huenei* YX V0003 sauropod. Arrow shows the anatomical location of the affected rib. Photograph by L.X.

**Figure 3:**
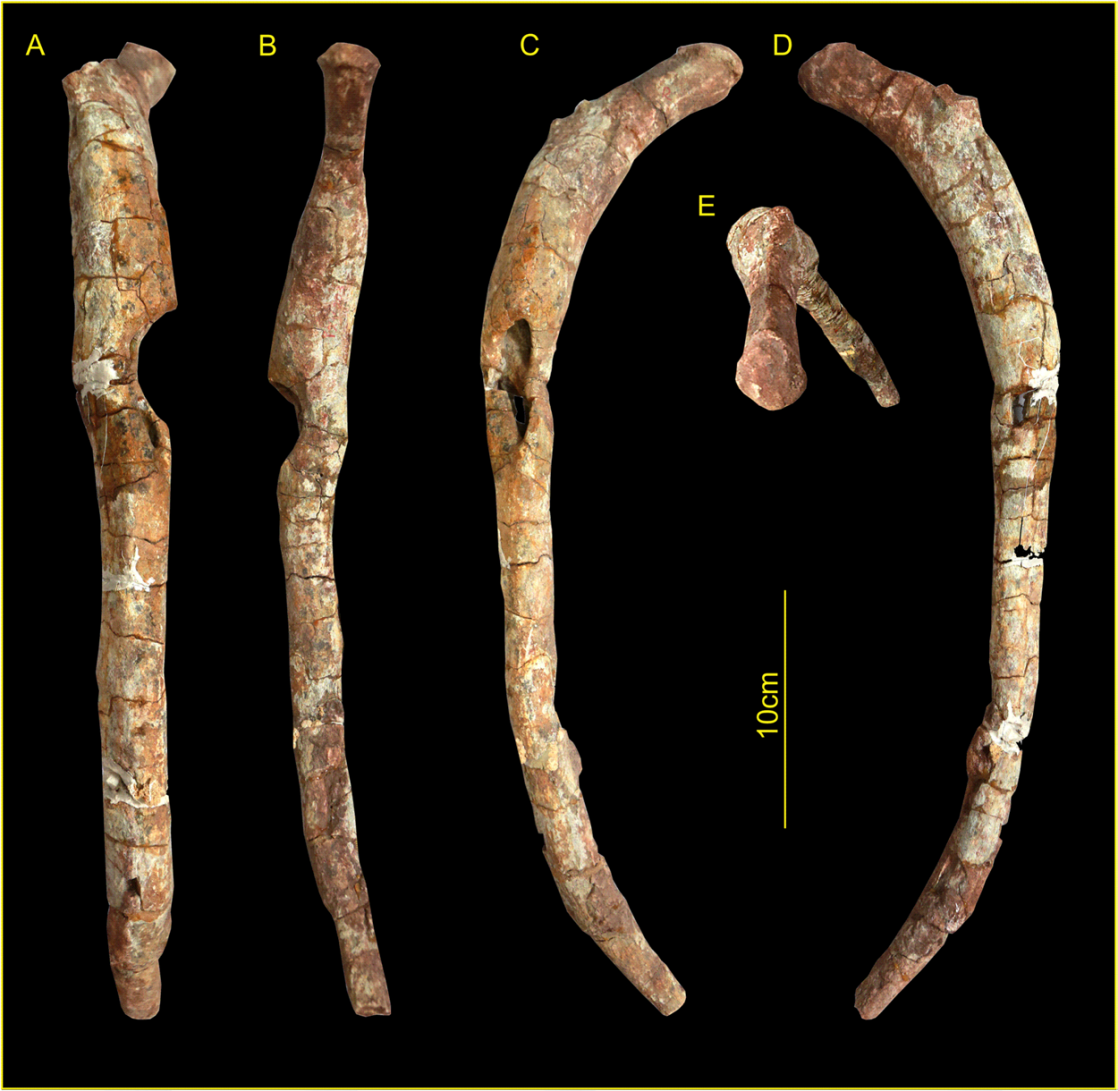
External morphology of the YX V0003 third rib. (**A**) lateral view; (**B**) medial view; (**C**) anterior (cranial) view; (**D**) posterior (caudal) view; (**E**) dorsal view. Scale bar is 10 cm. Photographs by L.X.

Only a single skeletal element showed clear evidence of skeletal pathology. The osseous abnormality is located on the third right rib of YX V003 (Fig. 2); no other osseous lesions or pathological traces were identified elsewhere on the partial skeleton. The rib is complete and has a chord length between the dorsal and ventral ends of 394 mm, with an overall circumferential (curve) length of 452 mm (Fig. 3).

In order to investigate the pathology affecting the specimen, the fossil was subjected to high-resolution imaging using micro-computed tomography (micro-CT). This non-invasive imaging technique has been demonstrated to provide a significant diagnostic advantage over conventional whole-bone morphoscopic or microscopic methods of imaging in cases of fossilised pathology^22,23^. Micro-CT imaging of the YX V003 rib was carried out using a Nikon Metrology XTH 225/320 LC dual source industrial micro-computed tomographic scanner, housed in the China University of Geosciences, Beijing (CUGB), China. Due to the size of the specimen in relation to the detector size and gantry size of the scanner, the lesion was imaged in two focussed portions split into a dorsal half (volume identifier [VI] XLD02) and a ventral half (VI XLD03) with a small zone of overlap between the two volumes of interest (VOI). Both VOI were scanned using a potential difference of 135 kV and a current of 67 µA, at an effective resolution of 37 µm; a TIFF-format image stack was generated in VG Studio Max following volume reconstruction. Further reconstruction by PSR-Q was undertaken using Avizo Amira 5.4 to generate both 2D orthoslice and 3D surface rendered views based on the volume data; multi-planar mode was used in Amira to allow the recovery of homologous orthoslices through the specimen for comparative purposes. Differential diagnosis based on established pathological and palaeopathological criteria was subsequently undertaken^24–27^.

## Description

The defect presents as a well-defined aperture which affects the lateral, medial and anterior surfaces of the rib (see Fig. 3A–C), and is located some 108 mm ventrally in the coronal plane (146 mm circumferentially) from the costovertebral junction. Viewed from the lateral and medial aspects, the defect presents as a semi-circular (concave) removal of bone, the loss of which extends almost half-way through the antero-posterior thickness of the shaft of the rib, to an approximate depth of 15.4 mm on the lateral surface. The defect is teardrop-shaped when viewed from the anterior aspect (Fig. 3C). The defect has a total external length of 54 mm, with the widest point some 20 mm from the blunted (dorsal end), reducing in width inferiorly to form a sharp tail at the ventral end of the aperture. The margin of the defect is largely intact and undamaged, although a comminuted block of cortical bone (15 mm long and 8.4 mm wide) has been lost on the opposing (medial/pleural) side of the rib (Fig. 3C,D), the margins of which clearly follow lines of post-fossilisation cracking – the loss of this area of bone is thus unrelated to the formation of the pathological defect. None of the fractures observed across the wider specimen are consistent with peri-mortem trauma or *in-vivo* failure, and instead present as transverse or right-angled breaks, showing block-comminution between major fracture blocks, and are thus ‘dry bone’ fractures and post-mortem/fossilisation in nature^28,29^. Many of these fracture boundaries have subsequently been infilled with calcite, particularly those following longitudinal split lines along the grain of the rib shaft.

**Figure 4:**
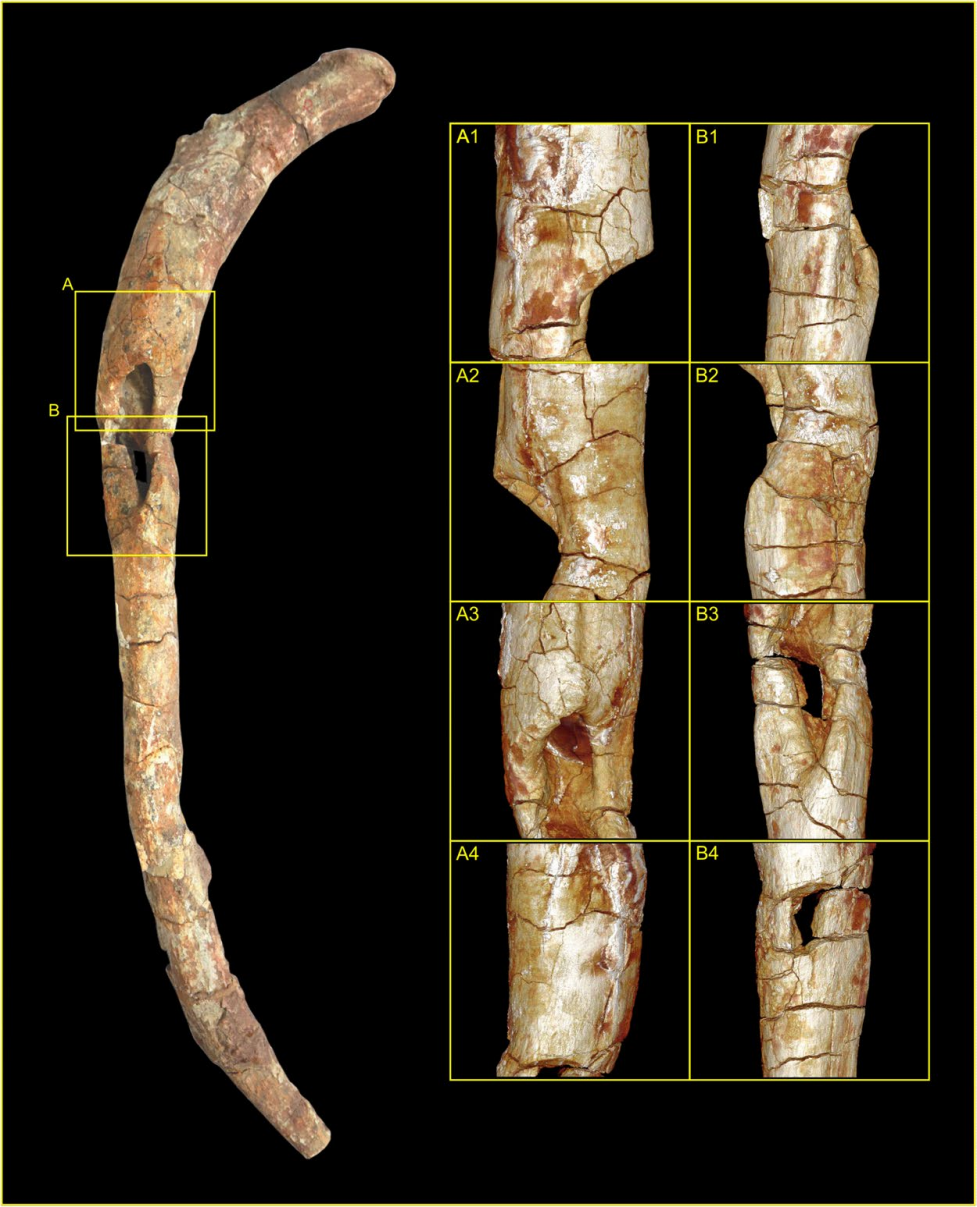
External morphology of the pathological *Lufengosaurus huenei* sauropod rib reconstructed from surface render of micro-computed tomographic (MCT) image volume. Schematic overlay of the photograph highlights the regions of interest (ROI) based on the respective MCT volumes ROIA and ROIB. Each ROI shows **(1)** lateral view; **(2)** medial view; **(3)** anterior (cranial) view; **(4)** posterior (caudal) view. Note, the section lines labelled A to F indicate the anatomical locations of MCT orthoslices presented in Fig. 5. Photograph by Lida Xing. Volume renders produced by P.S.R.-Q.

Externally, the margin of the defect is rounded, with clear softening and remodelling of the bone around the aperture (Fig. 4) – this creates a ridge which extends some 3 mm above the original bone surface. Where remaining cortical surface of the fossil survives intact, the surface is observed as irregular and slightly puffy, suggesting remnants of systematic sub-periosteal reaction or cortical remodelling, indicative of reactive new bone formation. No evidence of bone tumors, exostoses or spurs was observed.

**Figure 5:**
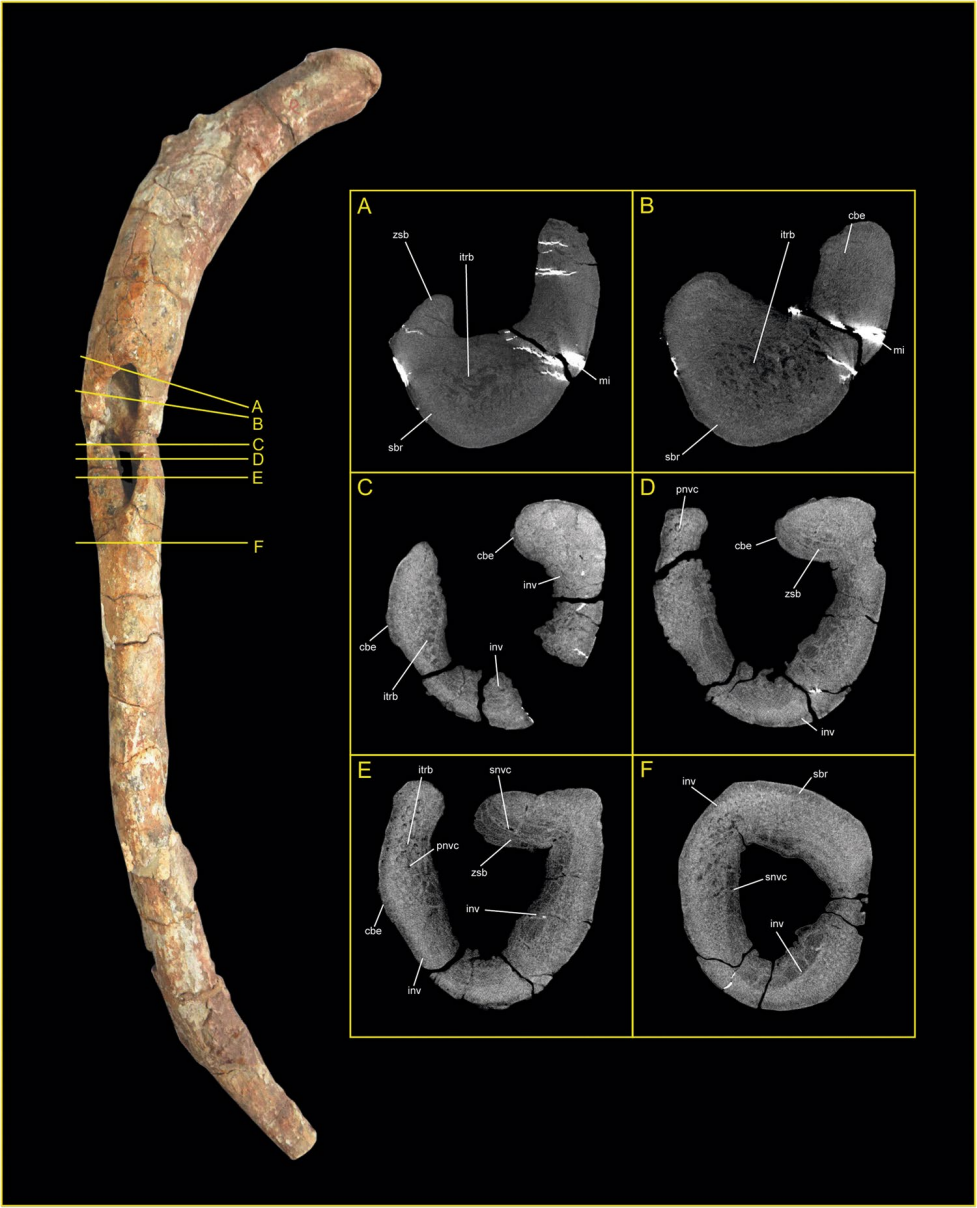
Transverse orthoslices produced from micro-computed tomography of the YX V0003 rib. Each slice represents an aligned transverse x-ray slice taken perpendicular to the cortical surface of the bone at the locations marked A to F in Fig. 4. Abbreviations: cbe = cortical bone expansion; inv = involcrum; itrb = internal trabeculae; pnvc = primary neurovascular canal; sbr = sequestered bone region; snvc = secondary neurovascular canal; zsb = zone of sclerotized bone. Photograph by Lida Xing. Orthoslices produced by P.S.R.-Q.

Internally, the defect comprises two distinct zones, separated by a convex bulge or island of bone (see Fig. 4A3,B3) coincidental with an external change of curvature of the rib shaft; there is clear evidence of remodelling and reactive bone formation surrounding the defect which extends into the medulla of the shaft. In cross-section, the defect and surrounding cortical bone present clearly defined zones of active surface and internal remodelling, with both exosteal and endosteal surfaces displaying areas of radiolucency indicative of reactive bone formation (Fig. 5A–F), which is present both as sequestra of necrotic bone and as involcrum formation. This contrasts with the presence of normal un-modified trabecular bone within the structure of the residual (normal) body of the shaft. Well-defined sclerotic zones are noted in cross-section, occurring extensively around the expanded margins of the defect and within the medula (Fig. 5A,D,E), and along the body of the shaft (where they are observed underlying zones of remodelling), indicating a quiescent condition with healing of the lesion.

## Differential diagnosis

Diagnosis was undertaken using both palaeopathological and clinical diagnostic criteria^24–27^ with clinical character recognition adapted for use in cases of micro-tomographic volume imaging^22,23,30^. The accumulated evidence for both osteolytic and osteosclerotic processes indicates that the disease process was both chronic (long-term) and clinically active at the time of death of the animal. The damage was associated with reactive new bone formation, and thus can be attributed to a phenomenon which occurred during the animal’s life^2,26,31^. Clear evidence of bone remodelling suggests that the dinosaur lived past the initial event that caused the damage to the osseous tissue. The limited distribution of the pathology rules out systemic diseases^1,26^. The position of the pathology (unassociated with a joint) also rules out consideration of gout, arthritis, or pseudarthrosis^25,26^. No other bioerosional bone damage was identified on the specimen, with any fractures or breaks post-mortem/fossilisation in nature.

A lytic lesion produced as a result of a bone infection is the most likely explanation due to the associated cortical bone loss and subsequent remodelling of the rib^2,31,32^, with osteomyelitis being suggested as the most parsimonious explanation. Osteomyelitis is a severe infection originating in the bone marrow, usually resulting from the introduction of pyogenic (pus-producing) bacteria into the bone; most commonly today this is staphylococcus, although streptococcus, salmonella, enterococcus and other organisms have been implicated as pathogenic vectors^33^. These pathogens can enter either via the bloodstream, or through wounds and open fractures which will introduce bacteria into the periosteum, cortex, marrow, and cancellous tissue. A distinctive feature of osteomyelitis in the skeleton is extensive new bone formation. This may totally encase the cortex of infected bone (sequestrum) as a shell of new bone, which is termed an involucrum. Sequestrated bone varies in size ranging from one or more small areas within an element to the entire diaphysis. Involucrum is the product of periosteal reaction to tissue necrosis, and although cortical bone may be dead as a consequence of infection, the periosteum may be viable and forms new bone to ensure continued normal function. Involucrum tends to be formed quickly and typically has an irregular surface. Present on the surface of the involucrum are often found large, circular openings or cloacae, which are channels of drainage for pus produced by the bacteria^33^. Such general features are evidenced in the YX V0003 specimen, supporting a diagnosis of osteomyelitis.

## Discussion

Osteomyelitis is a bacterial infection frequently distinguished by progressive inflammatory destruction of bone, in which the primary skeletal focus is in the bone marrow, and which thus occurs endosteally within the medulla. This is in contrast with osteitis, which primarily occurs within compact bone, and periostitis, which affects the outer layer of bone and its associated periosteal sheath. It is common for all three areas of bone to be involved in an infectious disorder, and determining the primary skeletal site of pathology may be difficult. In the case of YX V0003 the defect is localised and connects through to the medulla, providing both a focal site and a clear diagnosis. There are two primary categories of acute osteomyelitis: hematogenous osteomyelitis, caused by the seeding of the bacteria from a remote source, and direct or contiguous inoculation osteomyelitis, caused by direct contact of the tissue and bacteria during trauma^34^. In the case of YX V003, the loss of bone marrow with exposure of the medulla supports the hypothesis that direct osteomyelitis is the most likely process to have caused this bone pathology^2,31^. Reactive new bone in the form of periosteal reaction^35,36^ is a significant characteristic of bird or reptile osteomyelitis^27^. Other studies have also suggested that in modern mammals bone deformation can also be produced by osteomyelitis^37^. Long-term clinical symptoms include abrupt onset of high fever, local oedema, erythema, non-healing ulcers, sinus tract drainage, sepsis and tissue necrosis^33,38,39^.

Osteomyelitis has previously been observed in non-avian dinosaurs^2,8,26,40^ and attributed to, fractures and/or other injuries^2^. Reports of bone marrow infection (e.g., osteomyelitis) is rare in dinosaurs, especially Sauropodomorpha dinosaurs^2,32^. Our identification of osteomyelitis in a sauropodomorph is second only to that of García and colleagues^2^ who reported the pathology in a *Baurutitan britoi* from Argentina. The pathology reported here from *Lufengosaurus huenei* is the first recognition of an abscess in sauropods, and the third example of surviving osseous abnormalities or pathologies from the Yunnan area^1,9^. This discovery enriches the record of infections in dinosaurs, and extends our understanding of the frequency of paleopathology in Sauropodomorpha dinosaurs.

Given the physical location of the lesion within the thorax, and the relatively exposed nature of the costal margin to external insult, we suggest that the infection may have been initiated by a physical injury, such as a puncture or bite^26,33^. This suggests that the observed pathological processes arose as the result of a subsequently transferred (acquired) infective agent^32,41^. If so, the infective process would have been present for an extended period. If the infection extended in different directions with a similar speed^42,43^, the shape of the bone puncture would reflect the shape of the wound, in this instance a wide blunt opening with a sharpened end. The morphology of the damage suggests that the damage was produced as a result of a tooth or claw incision^44^. Whilst the large defect may be representative of a remodelled cloaca following localised necrosis (rather than a bite or puncture) we suggest that the size of the defect would preclude this. Such a large cloaca would likely produce a much greater degree of involucrum formation than is expressed here, and we consider it more likely that the primary area of bone loss represents a bite or tooth puncture as a consequence; the general tear-drop shape and overall pattern of the defect is in keeping with the morphology of bite-induced trauma and predatory behaviours recorded elsewhere in the dinosaur fossil record^5,45–48^.

No theropod skeletons have yet been recovered from the Fengjiahe Formation. However, the faunally-similar Lufeng formation has yielded four theropod genera *Sinosaurus*^9,49,50^, *Eshanosaurus*^51^, *Panguraptor*^52^. *Eshanosaurus* belongs to therizinosaur, which is considered to be herbivore^51,53^. *Panguraptor* are small coelophysids and may have not have the ability to attack *Lufengosaurus*. However, *Sinosaurus* is a famous and larger bodied predator, and would be able to attack *Lufengosaurus*^9,50^, and thus is a possible suspect for producing the injuries which resulted in the abscess and osteomyelitis. Whilst the exact identity of the proposed attacker is not critical, it is clear is that the attack was unsuccessful, and that the associated symptoms of the infection likely influenced the future life-history of the *Lufengosaurus*. Osteomyelitis is known to produce the fever, fatigue, nausea, loss of range of motion, tenderness, and discomfort, although necrotizing tracts of pasteurella bacteria extending to the brain have been reported in clinical cases in reptiles^54^, which are associated with elevated morbidity. Any or all of these symptoms would have negatively impacted the survival potential of the injured *Lufengosaurus* in the dynamic and complex ecosystems during the Jurassic.
